# Context-Specific Coordinately Regulatory Network Prioritize Breast Cancer Genetic Risk Factors

**DOI:** 10.3389/fgene.2020.00255

**Published:** 2020-03-26

**Authors:** Shuyuan Wang, Wencan Wang, Weida Wang, Peng Xia, Lei Yu, Ye Lu, Xiaowen Chen, Chaohan Xu, Hui Liu

**Affiliations:** College of Bioinformatics Science and Technology, Harbin Medical University, Harbin, China

**Keywords:** breast cancer, transcriptional factor, non-coding RNA, context-specific regulatory network, RWR algorithm

## Abstract

Breast cancer (BC) is one of the most common tumors, leading the causes of cancer death in women. However, the pathogenesis of BC still remains unclear, and the atlas of BC-associated risk factors is far from complete. In this study, we constructed a BC-specific coordinately regulatory network (CRN) to prioritize potential BC-associated protein-coding genes (PCGs) and non-coding RNAs (ncRNAs). We integrated 813 BC sample transcriptome data from The Cancer Genome Atlas (TCGA) and eight types of regulatory relationships to construct BC-specific CRN, including 387 transcription factors (TFs), 174 microRNAs (miRNAs), 407 long non-coding RNAs (lncRNAs), and 905 PCGs. After that, the random walk with restart (RWR) method was performed on the CRN by using the known BC-associated factors as seeds, and potential BC-associated risk factors were prioritized. The leave-one-out cross-validation (LOOCV) was utilized on the BC-specific CRN and achieved an area under the curve (AUC) of 0.92. The performances of common CRN, common protein–protein interaction (PPI) network, and BC-specific PPI network were also evaluated, demonstrating that the context-specific CRN prioritizes BC risk factors. Functional analysis for the top 100-ranked risk factors in the candidate list revealed that these factors were significantly enriched in cancer-related functions and had significant semantic similarity with BC-related gene ontology (GO) terms. Differential expression analysis and survival analysis proved that the prioritized risk factors significantly associated with BC progression and prognosis. In total, we provided a computational method to predict reliable BC-associated risk factors, which would help improve the understanding of the pathology of BC and benefit disease diagnosis and prognosis.

## Introduction

Breast cancer (BC), a type of cancer developing from breast tissue, is the most frequent occurrence and one of the leading causes of cancer-related deaths among women (Siegel et al., 2019). A large amount of study has been conducted to dissect the pathogenesis of BC, and multiple risk factors have been identified for the development of BC in the last decades. Extrinsic factors inclusive of dietary habits, long-term medical intervention, and carcinogens have been confirmed to commit the risk of BC (Kaminska et al., 2015). In particular, the inherent factors, including age, sex, race, genetic mutations, and disturbance of molecular pathways, contribute the most cases of BC. For example, epidemiological data demonstrated 50% of BCs occurred in women aged from 50 to 69 years, and both *BRCA1* and *BRCA2* mutations conferred a 60 to 80% lifetime risk for the development of BC (Matsen and Neumayer, 2013). Substantial single-nucleotide polymorphism (SNP) array screen revealed that *ESR1* gene amplification occurred in about 20% of BC patients (Holst et al., 2007). With the advances in RNA-sequencing techniques, non-coding RNAs (ncRNAs), especially microRNAs (miRNAs) and long non-coding RNAs (lncRNAs), are confirmed to be related with the pathology of BC (Bhan et al., 2017; Xu et al., 2017). For example, Yan et al. (2008) identified differentially expressed (DE) miRNAs in BC and suggested that miR-21 overexpression contributed to the poor prognosis of BC patients. Xu et al. (2017) identified a cluster of oncogenic upregulated lncRNAs in BC tissue and that the knockout of *DSCAM-AS1*, *TINCR*, or *HOTAIR* prohibited BC cell proliferation. Currently, several curated databases have archived the known BC-associated factors, such as the Comparative Toxicogenomics Database (CTD) (Davis et al., 2017), Human microRNA Disease Database (HMDD) (Huang et al., 2019), and LncRNADisease (Bao et al., 2019). Although great progress has been made in identifying genetic risk factors of BC, the genetic contribution to BC etiology remains to be elucidated (Skol et al., 2016).

Computational methods have been considered as effective means to decipher genetic risk factors for complex diseases (Oti et al., 2006; Natarajan and Dhillon, 2014; Chen et al., 2018a; Luo et al., 2019). For example, the guilt-by-association strategy is widely used to predict disease-associated genes in computational biology according to the phenomenon that genes participating in a common biological process tend to be correlated with similar phenotypes (Ideker and Sharan, 2008; Lee et al., 2011; Itzel et al., 2015). As an extension of this principle, functional and semantic similarity calculation between diseases and genes is employed to prioritize disease-related genes (Hu et al., 2017; Asif et al., 2018; Chen et al., 2018b). In addition, knowledge-based approaches are also developed to infer disease–gene associations. For example, Zhou and Skolnick (2016) provided a Know-GENE method to detect genes associated with given diseases by implementing a boosted tree regression approach which combined the gene–gene mutual information and known protein–protein interaction (PPI) networks. Network-based approaches are other types of frequently used methods for novel disease gene prediction. Kohler et al. (2008) implemented a random walk algorithm on the constructed PPI network to prioritize disease–gene associations. Sun et al. (2014) proposed a computational method to speculate potential human-disease-associated lncRNAs based on the lncRNA functional similarity network. Chen et al. (2015) provided two novel lncRNA functional similarity calculation models and introduced them into the model of Laplacian regularized least squares for disease–lncRNA relationship prediction. Vanunu et al. (2010) provided a network propagation method for prioritizing abnormal genes based on formulating constraints on the prioritization function, and protein complex associations also can be predicted. However, all these above methods do not consider the context-specific condition for disease genetic risk factor prediction. It is believed that if the context-specific status is provided, the biological associations will be constructed credibly. Furthermore, transcriptional and posttranscriptional coordinately regulatory networks (CRNs) have been demonstrated as powerful tools to establish biological associations, which could be employed to prioritize BC-associated risk factors (Wang et al., 2015, 2018).

In this study, we provided a computational method to prioritize BC-associated protein-coding and non-coding genes and compared the performance of a BC-specific CRN with other networks. Publicly available experimentally verified regulatory data and BC-associated high-throughput transcriptome data from The Cancer Genome Atlas (TCGA) were firstly integrated to construct a comprehensive BC-specific CRN, comprising TFs, miRNAs, lncRNAs, and protein-coding genes (PCGs). Then, the random walk with restart (RWR) algorithm was performed on the constructed CRN to prioritize BC-associated risk factors, using the known BC-associated factors as seeds. Leave-one-out cross-validation (LOOCV) proved the better performance of BC-specific CRN. Furthermore, differential expression analysis and survival analysis manifested that the prioritized factors were associated with BC onset and prognosis. In total, we constructed a BC-specific CRN and implemented a computational method to prioritize credible protein-coding and non-coding genes associated with BC, which would provide potential therapeutic targets for BC treatment.

## Materials and Methods

### Construction of Comprehensive BC-Specific CRN

The BC-specific CRN referred to the regulatory network that especially existed in the BC context. To fulfill this purpose, eight types of regulatory relationships among four types of factors (TFs, miRNAs, lncRNAs, and PCGs) and BC-associated high-throughput transcriptome data were integrated to form the BC-specific CRN.

First, eight types of regulatory relationships, incorporating TF–miRNA, TF–lncRNA, TF–PCG, miRNA–lncRNA, miRNA–TF, miRNA–PCG, lncRNA–TF, and lncRNA–PCG, were obtained from credibly curated databases and integrated to form the common CRN. TF-miRNA regulations were downloaded from TransmiR v2.0, a database recording manually surveyed experimentally supported TF regulations to miRNA (Wang et al., 2010). TF–lncRNA regulations were obtained from ChIPBase (Yang et al., 2013). Here, we only retained the credible TF–lncRNA regulations that presented in more than 20 datasets. Furthermore, we performed TRANSFAC MATCH programs to ensure lncRNA sequences possessing transcription factor binding sites (TFBS) (Matys et al., 2006). The final TF regulations to lncRNAs were gotten by integrating the ChIPBase data and TRANSFAC MATCH results. TF–PCG regulations were obtained from TRANSFAC (v12.4). miRNA–TF and miRNA–PCG regulations were integrated from two databases, miRecords (Xiao et al., 2009) and miRTarBase (Chou et al., 2018). We obtained the union set of the relationships existing in these two datasets. miRNA–lncRNA regulations were obtained from LncBase v2 (Paraskevopoulou et al., 2016). We obtained the interactions provided in the experimental module, and the prediction score should be no less than 0.95. lncRNA–TF and lncRNA–PCG regulations were downloaded from LncReg and LncRNA2Target (v2.0) (Jiang et al., 2015). We retained the union set of lncRNA regulations to TFs and PCGs presented in two databases. Incorporating all the above regulations, we finally got the common CRN, which comprised candidate BC-specific regulatory relationships.

Next, we derived specifically highly expressed and co-expressed regulatory relationships in the BC context to obtain a BC-specific CRN. The BC-associated high-throughput TF, miRNA, and PCG expression profiles were collected from TCGA, and the lncRNA expression profiles were derived from The Atlas of Non-coding RNAs in Cancer (TANRIC). The intersection samples having the expression profiles of all these four types of factors were retained. Highly expressed genes are defined as those whose expression are ranked in the top 50% of all genes in more than 50% samples. Co-expressed relationships are defined as relationships whose Pearson’s correlation coefficient (PCC) values (or absolute PCC values) are ranked in the top 20% of all highly expressed genes’ pairwise PCC values calculated from each type of regulatory relationship. For TF–miRNA, the PCC values of all the couples of highly expressed TFs and miRNAs were calculated. Then the TF–miRNA pairs ranked in the top 20% of all these PCC values were retained. Similarly for the TF–lncRNA and TF–PCG, the pairs having PCC values that are top 20% ranked were retained. For miRNA–lncRNA, miRNA–TF, miRNA–PCG, the pairs having PCC values that are bottom 20% ranked were retained. For lncRNA–TF and lncRNA–PCG, the pairs having absolute PCC values that are top 20% ranked were retained. All these retained pairs intersecting with the common CRN constituted the final BC-specific CRN.

In addition, we constructed the PPI network to compare the performance of the CRN. The PPI relationships were obtained from the STRING database (Szklarczyk et al., 2019). We retained the relationships with a direct evidence score of >0.9 to form the common PPI network. The BC-specific PPI network was obtained by retaining the common PPI network relationships exhibiting high expression and co-expression in the BC context, which were calculated as described above.

### Collection of Known BC-Associated Factors

The known BC-associated TFs, miRNAs, lncRNAs, and PCGs were obtained from publicly available data resources. BC-associated TFs and PCGs were obtained from CTD, and the TFs and PCGs with direct evidence to the BC were retained. Next, we downloaded the BC-associated miRNAs from HMDD v3.0 (Huang et al., 2019). The BC-associated lncRNAs were integrated from LncRNADisease v2.0 (Bao et al., 2019) and Lnc2Cancer v2.0 (Gao et al., 2019), both of which were curated databases for disease-associated lncRNAs. We integrated the union of these two lncRNA sets as known BC-associated lncRNAs. All these obtained BC-associated TFs, miRNAs, lncRNAs, and PCGs were mapped to the BC-specific CRN, and the intersection nodes were used as seeds for the RWR algorithm.

### Prioritization of Potential BC-Associated Risk Factors With RWR

We thus conducted an RWR method on the BC-specific CRN to prioritize potential BC-associated risk factors. Here, the obtained known BC-associated factors were employed as seed nodes. We denoted *S*_0_ as the initial score vector and *S*_t_ as a process vector in which the *i*th element represented the probability of the random walker appearing at node *i* in step *t*. We let α measure the restart probability of the random walk at the initial nodes in each step. Also *P* represented the probability transition matrix (PTM), and it was obtained from the adjacency matrix of the BC-associated CRN. The formula is described as

$$p\,\left(i,j\right)=\left\{ \begin{array}{cc} M\,\left(i,j\right)/\sum_{\text{j}}M\,\left(i,j\right), & i\,f\,\sum_{\text{j}}M\,\left(i,j\right)\neq 0\\ 0, & o\,t\,h\,e\,r\,w\,i\,s\,e \end{array}\right.$$

where *p*(*i*, *j*) is the entry in the PTM and *M* (*i*, *j*) is the entry in the adjacency matrix. The score vector in step *t* + 1 can be defined as follows:

$$S_{t+1}=\left(1-\alpha \right)\,P\,S_{\text{t}}+\alpha \,S_{0}$$

Here, the restart probability α was set as 0.5, and the initial score *S*_0_ of each seed node was set as 1/*n* (where *n* was the number of total seed BC-associated factors). The initial scores of all other nodes were set as 0 (Li and Patra, 2010; Chen et al., 2016). It is natural that the score of each node will become stable with the iteration steps going on. We set the stable scores as *S*_∞_ when the difference between *S*_t_ and *S*_t__+__1_ was no more than 10^–10^. Then the final stable scores *S*_∞_ could be used to measure the proximity of each node to the seed nodes. Thus, all candidate nodes in the BC-specific CRN could be ranked based on *S*_∞_, and the top-ranked nodes could be speculated to be closely related with BC.

### Functional Analysis for Predicted BC-Associated Risk Factors

We conducted a functional analysis for the putative BC-associated risk factors. We first extracted the top-100-ranked potential BC-associated risk factors (excluding seeds), inclusive of TFs, miRNAs, lncRNAs, and PCGs, and conducted functional enrichment analysis separately. We employed DAVID to conduct gene ontology (GO) and Kyoto Encyclopedia of Genes and Genomes (KEGG) pathway enrichment analysis for the obtained TFs and PCGs separately (Huang da et al., 2009). For the obtained miRNAs, we firstly gathered the experimentally verified miRNA targets from miRecords (Xiao et al., 2009) and miRTarBase (Chou et al., 2018); then all these miRNA targets underwent GO and KEGG pathway enrichment analysis by DAVID. In addition, for the obtained lncRNAs, we extracted associated TFs and PCGs for each obtained lncRNA from ChIPBase (Yang et al., 2013), LncReg (Zhou et al., 2015), and LncRNA2Target (v2.0) (Jiang et al., 2015), and the union set of obtained TFs and PCGs was inputted into DAVID to perform functional enrichment analysis. Furthermore, GO enrichment analysis was conducted for the known BC-associated TFs, miRNAs, lncRNAs, and PCGs separately, as described above. Then the union set of these significant GO categories was regarded as the BC-associated GO terms. We adopted the same criteria for all these functional analyses, in which GO analysis employed the biological process (BP) category and the significant level was set at *P* < 0.05. In the end, we computed the functional similarity scores between the GO terms enriched in top-ranked BC-associated factors and the BC-associated GO terms. The calculative process was conducted by using the GOSemSim R package (Yu et al., 2010). The widely used “Lin” parameter was assigned to compute the two given GO terms’ semantic similarity, and the rcmax method was used as a combined method to accumulate multiple GO terms. We also conducted 1,000 random tests to assess the significance of obtained functional similarity scores. The same number of GO terms as the real situation was randomly chosen in each random test, and the functional similarity scores between the random GO term set and the BC-associated GO terms were calculated. The *P*-value was computed as the ratio of stochastic functional similarity scores higher than the true functional similarity score.

### Differential Expression Analysis

TF, miRNA, and PCG expression data were generated by next-generation sequencing, and the read count data could be available from TCGA. Here, we dealt with the TFs and PCGs together and used genes to refer to them both. Based on read counts, we used the edgeR package and calculated fold change (FC) to derive DE genes and miRNAs (Robinson et al., 2010). The paired BC samples in TCGA were retained, and genes and miRNAs with <1 count per million (CPM) in more than half of the samples were filtered out. Then, we used the exactTest function to implement an exact test for the genes and miRNAs. The significantly DE genes and miRNAs were obtained by selecting those genes and miRNAs with an adjusted *P*-value < 0.05 and | log2FC| > 1. The paired BC samples with lncRNA expression data were extracted from TANRIC. Because expressions of lncRNA were presented in an RPKM unit and were normalized to follow a normal distribution, a linear model was fitted for each lncRNA by using the lmFit function of the R package limma (Ritchie et al., 2015). Then the eBayes function was used to implement an empirical Bayes method to rank lncRNAs for differential expression. The significantly DE lncRNAs were obtained by selecting those with an adjusted *P*-value < 0.05 and | log2FC| > 1.

### Survival Analysis

The univariate Cox regression analysis was performed to assess the association between the prognosis of survival and the putative BC-associated risk factors. A risk score formula was implemented to measure the contribution of the predicted BC-associated risk factors to the survival of BC patients, which was computed from the linear integration of the expression values and the regression coefficient obtained from the univariate Cox regression analysis. The detailed formula was described as follows.

$$S\,c\,o\,r\,e_{\text{Risk}}=\sum_{i=1}^{\text{n}}r_{\text{i}}\times \mathrm{Exp}\,\left(x_{\text{i}}\right)$$

where *r*_i_ represents the univariate Cox regression coefficient of the predicted BC-associated factor *i* and *n* is the top-ranked number for factors we prioritized (100 assigned here). Exp(*x*_i_) represents the expression value of factor *i* in the corresponding patient. We used the median risk score as a cutoff to classify patients into low-risk and high-risk groups. The Kaplan–Meier survival analysis was performed for these two groups, and statistical significance was evaluated using the log-rank test. All analyses were performed by the R package “survival” within the R framework. The coxph function was used to obtain the univariate Cox regression coefficient of the predicted BC-associated factor, and the survdiff function was used to perform a log-rank test.

## Results

### Construction and Characterization of the BC-Specific CRN

**FIGURE 1:**
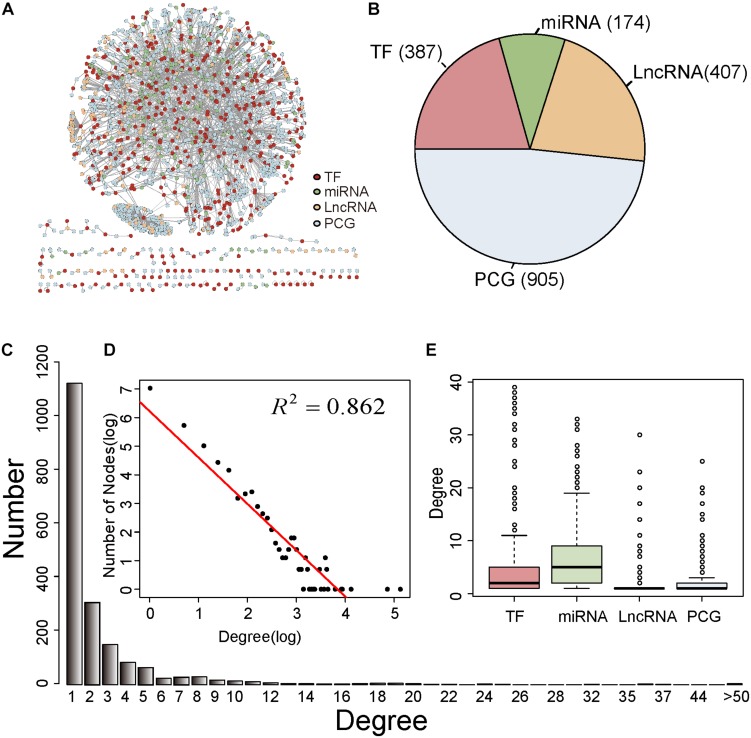
Characteristics of the BC-specific CRN. **(A)** Overview of the BC-specific CRN. The TFs, miRNAs, lncRNAs, and PCGs were red, green, yellow, and blue colored. **(B)** The proportion of TFs, miRNAs, lncRNAs, and PCGs in the CRN. **(C)** Degree distribution of all nodes in the CRN. **(D)** The log-log plots for the degree distributions of all nodes in the CRN **(E)** Degree distributions for TFs, miRNAs, lncRNAs, and PCGs in the CRN.

In this study, we firstly integrated experimentally verified regulatory relationships from publicly available data resources to obtain a common CRN (Supplementary Table S1). When combining the transcriptome data in BC, we constructed a BC-specific CRN (section “Materials and Methods,” Supplementary Table S2). The BC-specific CRN included 2,582 edges, comprising 387 TFs, 174 miRNAs, 407 lncRNAs, and 905 PCGs (Figures 1A,B). We inspected the degree distribution of the network to get an overview of the CRN. As shown in Figure 1C, most nodes (58.9%) of the CRN had one degree and only few nodes had a high degree. Next, the power law distribution of the form *y* = 504.1 × 10^–1.62^ (*R*^2^ = 0.862) was fitted for the whole degree of the BC-specific CRN (Figure 1D). This illustrated that the CRN met scale-free topology, which was a common feature for most biological networks (Barabasi and Oltvai, 2004). In addition, we investigated the degree distributions for TFs, miRNAs, lncRNAs, and PCGs (Figure 1E). The miRNAs had a higher median degree than other factors, which meant that miRNAs were likely to act as hubs in the BC-specific CRN.

### Performance Evaluation

**FIGURE 2:**
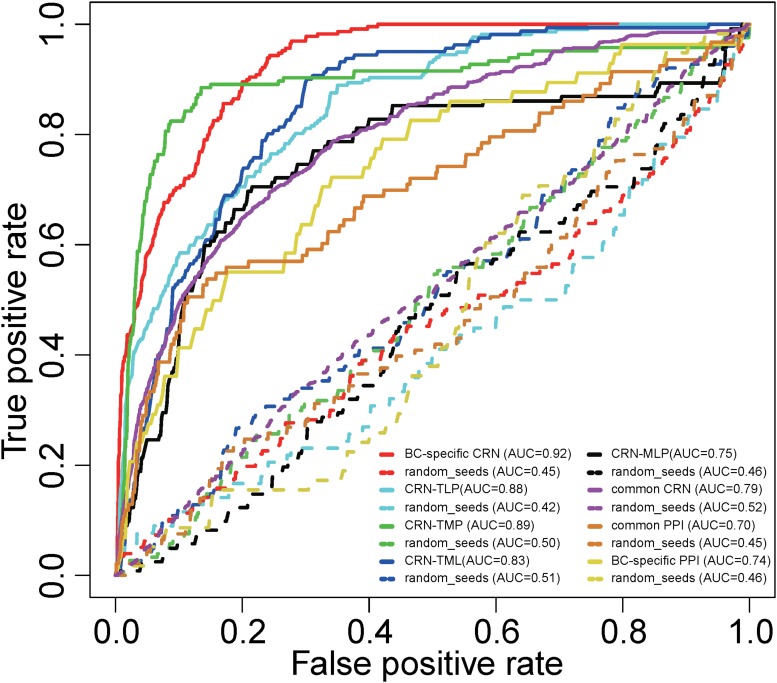
Receiver operating characteristic curves and AUC values for the RWR method on the whole and partial BC-specific CRNs, common CRN, BC-specific PPI network, and common PPI network with real seeds and random seeds.

To assess the performance of our method for inferring potential BC-associated risk factors, we conducted a LOOCV analysis. The known BC-associated factors from curated databases were integrated, and 1,298 credible BC-associated factors were obtained in total. When mapping these factors to the BC-specific CRN, we finally got 177 BC-associated factors as seeds, including 49 TFs, 95 miRNAs, 15 lncRNAs, and 18 PCGs (Supplementary Table S3). Each known BC factor was left out in turn as the test case, and the other known BC factors were taken as seeds. All the other nodes in the BC-specific CRN were regarded as candidate BC-associated factors. Then different sensitivities and specificities were calculated by varying the threshold. Finally, a receiver operating characteristic (ROC) curve was plotted, and the value of the area under the curve (AUC) was calculated. Our proposed method tested on known BC-associated factors achieved an AUC of 0.92 (Figure 2), demonstrating excellent performance. Here, the BC-specific CRN included four kinds of factors (TFs, miRNAs, lncRNAs, and PCGs) and eight types of regulations (TF–miRNA, TF–lncRNA, TF–PCG, miRNA–lncRNA, miRNA–TF, miRNA–PCG, lncRNA–TF, and lncRNA–PCG). In order to evaluate the effectivity and reliability of the BC-specific CRN, we compared the performance of partial CRN. The AUCs were calculated for the CRN-TLP network (TFs, lncRNAs, and PCGs only), CRN-TMP network (TFs, miRNAs, and PCGs only), CRN-TML network (TFs, miRNAs, and lncRNAs only), and CRN-MLP network (miRNAs, lncRNAs, and PCGs only) separately, by performing LOOCV. As shown in Figure 2, the AUCs were 0.88, 0.89, 0.83, and 0.75 for the CRN-TLP, CRN-TMP, CRN-TML, and CRN-MLP networks, respectively, which were lower than those using the whole CRN. We also evaluated the comprehensiveness and accuracy of seeds used in the RWR. The seeds were randomly chosen from candidate nodes for all these five networks, and we calculated the AUC values by performing LOOCV as above. The AUC values under randomization tests were much lower than those in real situations (0.45, 0.42, 0.50, 0.51, and 0.46) (Figure 2). In addition, we detected the performance of the common CRN, common PPI network, and BC-specific PPI network, and as shown in Figure 2, their AUCs were 0.79, 0.74, and 0.70, respectively. The result indicated that the CRN performed better than did the PPI, and BC-specific PPI was better than common PPI. All these results confirmed that the BC-specific CRN with known BC seeds is valid and reliable for BC-associated risk factors.

### Identification of BC-Associated Risk Factors

We finally prioritized potential BC-associated risk factors by performing the RWR method on the BC-specific CRN. The prioritizations of all candidate BC-associated risk factors were provided in Supplementary Table S4. The top-100-ranked candidate risk factors, including 48 TFs, 2 miRNAs, 14 lncRNAs, and 36 PCGs, were further validated by literature mining, in which 71 factors had been verified to be associated with BC in recently published articles (Supplementary Table S5). For example, the first-ranked factor *MYC* was recently reported to be upregulated by hematological and neurological expressed 1 (*HN1*) in BC and thus promoted the progression of BC (Zhang et al., 2017). The TF risk factor *SP1* was demonstrated to upregulate the known BC-associated lncRNA *TINCR*, which in turn stimulated cell proliferation of BC (Liu et al., 2018). The top-ranked *miR-365* expression level was found to be significantly higher in BC tissues, and the relatively high expression levels promoted cell proliferation and invasion in BC by targeting the known BC-associated PCG *ADAMTS-1* (Li et al., 2015). The lncRNA *OIP5-AS1* was recently demonstrated to play a critical role in promoting BC progression through acting as a *miR-129-5p* sponge to upregulate the expression of *SOX2* (Zeng et al., 2019). The top-ranked PCG *VEGFA*, involved with *miR-205* and *FGF2*, contributed to the resistance to chemotherapeutics in BC, which promoted the BC progression and suppressed cell apoptosis (Hu et al., 2016). Another top-ranked PCG *BCL2L11* was involved in tamoxifen response of BC by disturbing the expression levels of cleaved *PARP* and *caspase-3*, which would affect BC prognosis (Yin et al., 2017). The extensive literature survey exhibited the feasibility of our method to predict BC-associated risk factors.

### Functional Characteristics of Predicted BC-Associated Risk Factors

**FIGURE 3:**
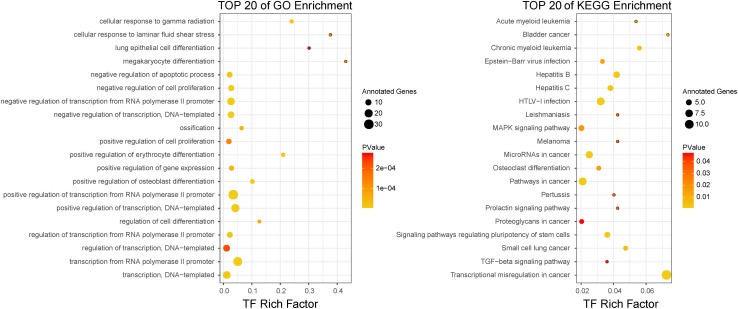
The top 20 GO enrichment results and KEGG enrichment results for top-ranked TFs.

The top-100-ranked candidate BC-associated risk factors then underwent functional analysis separately (see section “Materials and Methods” for details). For the top-ranked TFs, the top 20 significantly enriched GO terms and KEGG pathways were shown in Figure 3. We observed that some cancer-related GO terms, such as *positive regulation of cell proliferation* and *negative regulation of apoptotic process*, were enriched in these top-ranked TFs. Some significantly enriched KEGG pathways were also associated with cancers, for instance, *pathways in cancer* and *MAPK signaling pathway*. In addition, some other cancer-related pathways, such as *small cell lung cancer*, *bladder cancer*, and *melanoma* were also enriched in top-ranked TFs. In accordance with the functions of TFs, multiple transcription-related GO terms and KEGG pathways were enriched in the top-ranked TFs. Similar to top-ranked TFs, the cancer-related GO terms and KEGG pathways, such as *cell proliferation* and *pathways in cancer*, were also enriched in top-ranked miRNAs, lncRNAs, and PCGs.

In order to further demonstrate that the top-ranked factors were related with BC, we compared the GO terms enriched by the top-100-ranked BC with those enriched by known BC-associated factors. The numbers of overlapped enriched GO terms among the top-ranked factors and known BC-associated factors were high (Supplementary Figure S1A). We computed the functional similarity scores between the BC-related GO terms and the top-100-ranked factors enriched GO terms. The functional similarity scores between the BC-related GO terms and those enriched by top-ranked TFs, miRNAs, lncRNAs, and PCGs were 0.973, 0.968, 0.977, and 0.984, respectively (Supplementary Figure S1B). The random functional similarity scores for each kind of factors, which were calculated by randomly choosing the same number of GO terms as the true situation, were significantly lower than the real scores (Supplementary Figure S1B). The results showed that all these *P*-values were less than 2.2 × 10^–16^, which demonstrated that the top-ranked factors were significantly associated with BC. The functional characteristics of the top-ranked factors indicated that our method was capable of identifying novel BC-associated factors.

### Prioritized BC-Associated Risk Factors Are Potential Prognostic Biomarkers

**FIGURE 4:**
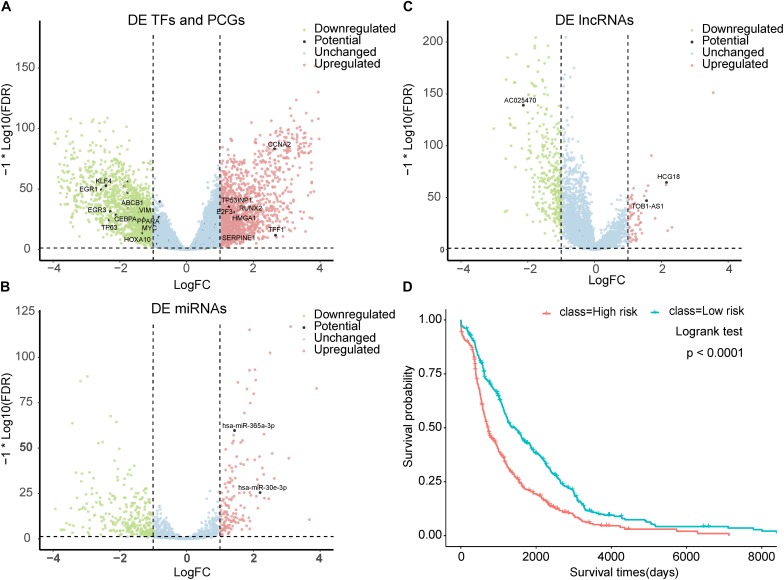
Prioritized BC-associated factors are potential prognostic biomarkers. **(A)** Volcano plot for the DE genes of BC. The top-ranked TFs and PCGs that differentially expressed were marked. **(B)** Volcano plot for the DE miRNAs of BC. The top-ranked miRNAs that differentially expressed were marked. **(C)** Volcano plot for the DE lncRNAs of BC. The top-ranked lncRNAs that differentially expressed were marked. **(D)** Kaplan–Meier analysis for overall survival of patients with high-risk or low-risk scores. *P*-value was calculated using the two-sided log-rank test.

We characterized the expression status of the top-100-ranked BC-associated risk factors in BC. The 224 paired BC samples and 15,591 genes with a CPM > 1 in at least half of the samples were obtained from TCGA. Differential expression analysis based on read counts was performed by using the edgeR package. At a significance level of an adjusted *P*-value < 0.05 and | log2FC| > 1, we identified 3,000 significantly DE genes, with 1,495 upregulated and 1,505 downregulated in BC (Figure 4A and Supplementary Table S6). The top-100-ranked BC-associated TFs and PCGs (48 TFs and 36 PCGs) were compared with the DE genes. There were 18 TFs and 15 PCGs exhibiting DE (Figure 4A) and hypergeometric test *P*-values of 1.62 × 10^–3^ and 2.42 × 10^–3^ separately. Furthermore, we also detected the expression status of the known BC-associated genes. A total of 555 known BC-associated genes were collected, and 161 genes were DE. The hypergeometric test *P*-value was 1.04 × 10^–8^. We retained 206 paired BC samples and 268 miRNAs with a CPM > 1 in at least half of the samples from TCGA. We identified 86 significantly DE miRNAs (Figure 4B) in total, and the two miRNAs in top-100-ranked BC-associated factors were both DE miRNAs. The hypergeometric test *P*-value was 1.09 × 10^–3^. A total of 546 known BC-associated miRNAs were obtained, and 74 miRNAs were DE. The hypergeometric test *P*-value was <2.2 × 10^–16^. The 210 paired BC samples with 12,727 lncRNA expression data were obtained from TANRIC. By using the criteria described in the “Materials and Methods” section, we identified 357 DE lncRNAs (Figure 4C). The 14 lncRNAs in top-100-ranked BC-associated factors embraced three lncRNAs exhibiting DE, and the hypergeometric test *P*-value was 6.33 × 10^–3^. A total of 146 known BC-associated lncRNAs were obtained, and 12 lncRNAs were DE. The hypergeometric test *P*-value was 7.39 × 10^–4^. Furthermore, we depicted remarkable top-100-ranked TFs, miRNAs, lncRNAs, and PCGs that expressed differentially in BC (Figures 4A–C).

In addition, we assess the clinical relevance of these predicted BC-associated risk factors. BC patients’ survival data and transcriptome data were obtained from TCGA. In total, we obtained 832 BC samples and then conducted a survival analysis on these patient samples. We firstly performed the univariate Cox regression analysis for each predicted BC-associated factor and obtained a univariate Cox regression coefficient for each factor. Then a risk score was computed for each BC patient by linear integration of the expression data and Cox regression coefficient of predicted BC-associated risk factors (see section “Materials and Methods” for details). According to the median risk score, all these BC patients were separated into a low-risk group (416 patients) and high-risk group (416 patients). The Kaplan–Meier survival analysis was conducted for the two groups, and the log-rank test *P*-value was less than 1.0 × 10^–3^ (Figure 4D). All these results indicated that the predicted BC-associated factors could potentially serve as prognostic biomarkers for BC.

## Discussion

Breast cancer is the most common malignancy in women worldwide with differing molecular signatures, prognoses, and responses to therapies (Siegel et al., 2019). Although great progress has been achieved in identifying risk factors of BC development in the last decades, the comprehensive landscape of genetic contribution to BC etiology remains to be further elucidated (Skol et al., 2016; Sun et al., 2017). In addition, the identification of novel BC risk factors is beneficial for BC-targeted therapy, which represents a promising strategy for BC treatment. A context-specific regulatory network, which provides a general view of the transmission of genetic information and characterizes the concrete biological status, has been proven as a powerful tool for studying biological issues (Wang et al., 2015, 2018). The constructed BC-specific CRN and computational method presented here prioritized BC-associated protein-coding and non-coding genes, exhibiting high credible performance.

The landscape of CRN has been described elaborately in the past decades (Liang et al., 2012; Wang et al., 2015). However, exhaustive regulatory associations still need further investigation. Especially, the depiction of lncRNA regulations to TFs and PCGs is still at a preliminary level (Kopp and Mendell, 2018). Furthermore, competing endogenous RNA (ceRNA) relationships that existed in TFs, miRNAs, lncRNAs, and PCGs lead to further complicated regulations among these factors, which should be taken into consideration in future analyses of CRN (Tay et al., 2014). It also should be noted that the algorithm provides potential associations rather than suggesting causality. Further experiment confirmation is needed to clarify the BC pathogenesis. Furthermore, BC can be categorized into different subtypes based on the immunohistochemical analysis of the molecular markers, such as basal-like, HER2+, luminal A, and luminal B. Single-cell RNA sequencing can categorize the BC subtypes in more detail (Sun et al., 2017). In general, different BC subtypes possessed distinct genetic risk factors. With the abundance of research for the BC subtype analysis, we will be able to prioritize subtype-specific risk factors and provide more comprehensive information for BC pathogenesis. In summary, we constructed a BC-specific CRN which could characterize the complex regulatory relationships of BC and serve as an effective tool to predict BC risk factors, which was enlightening for other disease gene prioritization.

## Data Availability Statement

Publicly available datasets were analyzed in this study. This data can be found here: https://portal.gdc.cancer.gov/.

## Author Contributions

HL and SW: conceptualization and funding acquisition. SW, WenW, and WeiW: formal analysis. PX, LY, and YL: data curation. SW and WenW: investigation. HL and XC: methodology. HL, XC, and CX: supervision. WenW: visualization. SW: writing – original draft. HL and CX: writing – review and editing.

## Conflict of Interest

The authors declare that the research was conducted in the absence of any commercial or financial relationships that could be construed as a potential conflict of interest.

## Supplementary Material

FIGURE S1 Prioritized factors are function associated with BC. **(A)** The intersection diagram of prioritized factors and BC-associated GO terms. **(B)** Distribution of random functional similarity scores for top ranked TFs, miRNAs, lncRNAs, PCGs and BC-associated GO terms. The triangles indicate the true functional similarity score for top ranked TFs, miRNAs, lncRNAs and PCGs BC-associated GO terms.

TABLE S1 The list of common CRN.

TABLE S2 The list of BC-specific CRN.

TABLE S3 The list of 177 seed BC factors.

TABLE S4 Prioritization of all candidate factors.

TABLE S5 The top 100 ranked candidate BC-associated factors validated by recently published articles.

TABLE S6 The list of significantly DE TFs, PCGs, miRNAs and lncRNAs in BC (adjusted *P*-value < 0.05 and | log2FC| > 1).
