# Supplementary figures and images for: Context-Specific Coordinately Regulatory Network Prioritize Breast Cancer Genetic Risk Factors
Source: Front Genet. 2020 Mar 26;11:255. doi: 10.3389/fgene.2020.00255 (PMC7113376; doi:10.3389/fgene.2020.00255)

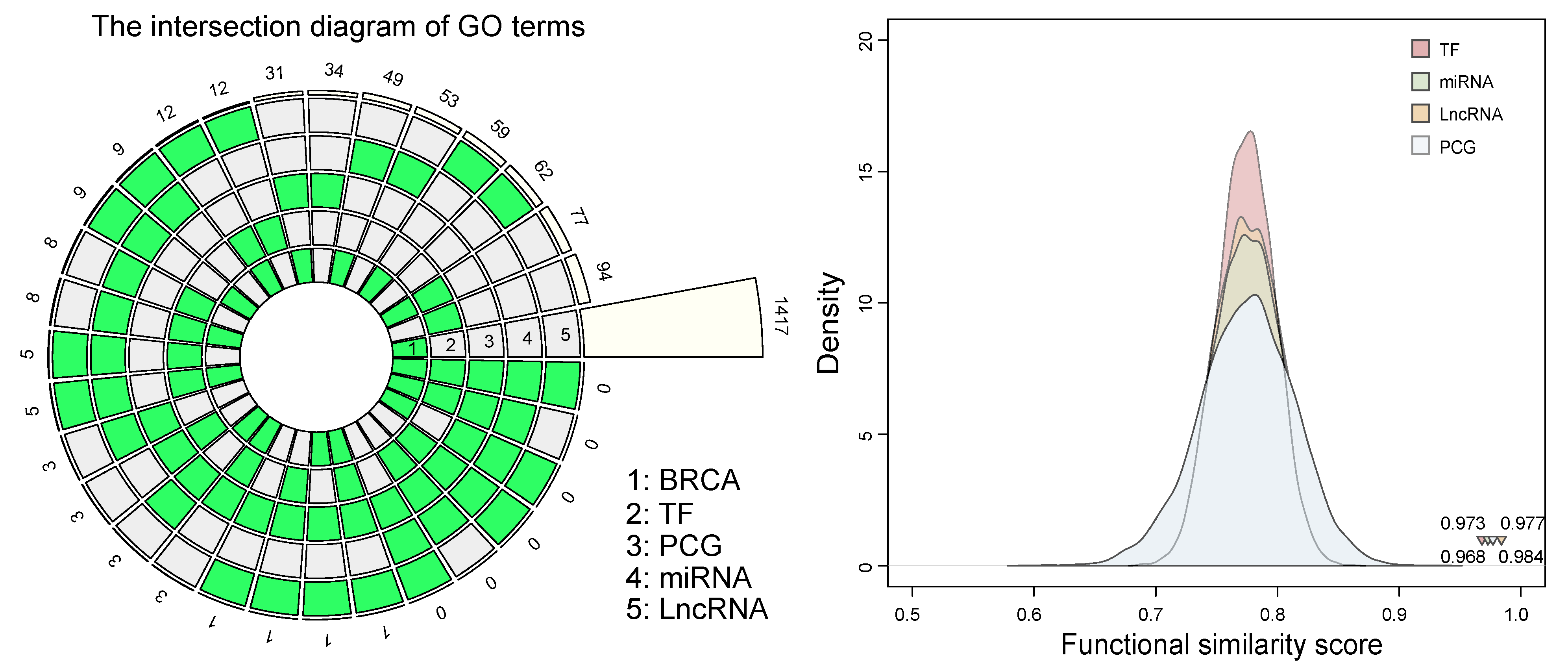

Supplement: FIGURE S1 — Prioritized factors are function associated with BC. (A) The intersection diagram of prioritized factors and BC-associated GO terms. (B) Distribution of random functional similarity scores for top ranked TFs, miRNAs, lncRNAs, PCGs and BC-associated GO terms. The triangles indicate the true functional similarity score for top ranked TFs, miRNAs, lncRNAs and PCGs BC-associated GO terms. [file Image_1.TIF]
